# Possible Macrophage Activation Syndrome Caused by Endoscopic Retrograde Cholangiopancreatography for Bacteremia Due to Chronic Cholelithiasis

**DOI:** 10.7759/cureus.30932

**Published:** 2022-10-31

**Authors:** Hiroyuki Furuta, Yudai Tanaka, Tsuyoshi Mishiro, Chiaki Sano, Ryuichi Ohta

**Affiliations:** 1 Family Medicine, Shimane Medical University, Izumo, JPN; 2 Commnity care, Unnan City Hospital, Unnan, JPN; 3 Internal Medicine, Unnan City Hospital, Unnan, JPN; 4 Community Medicine Management, Shimane University Faculty of Medicine, Izumo, JPN; 5 Community Care, Unnan City Hospital, Unnan, JPN

**Keywords:** general medicine, rural hospital, endoscopic retrograde cholangiopancreatography, macrophage activation syndrome, occult bacteremia, illness-related corticosteroid insufficiency, hypoglycemia

## Abstract

Hypoglycemia is caused by various clinical diseases. Among older patients, occult bacteremia may cause critical illness-related corticosteroid insufficiency (CIRCI), triggering hypoglycemia. Additionally, older patients have various chronic medical and homeostatic conditions. Interventions may be needed when chronic conditions cause clinical diseases and CIRCI. Herein, we report a case of possible macrophage activation syndrome (MAS) caused by endoscopic retrograde cholangiopancreatography for bacteremia due to chronic cholelithiasis in an 85-year-old man. Interventions for chronic conditions could impinge on homeostasis in older patients, causing acute conditions such as MAS. Among older frail patients with chronic conditions, interventions for chronic conditions should be discussed, including the triggering of other acute conditions, such as MAS.

## Introduction

Hypoglycemia is caused by various critical conditions, especially in older patients, and should be detected promptly to improve their condition. Insulin and corticoids in the body control glycolysis and the secretion of these hormones can be impaired by various critical diseases, such as sepsis, adrenal insufficiency, and malignancies [1]. Sepsis can affect glucose metabolism in the body, leading to an imbalance between the need and demand for glucose in peripheral tissues [2]. Adrenal insufficiency is prevalent among older patients with various diseases [3]. Malignancy can also cause hypoglycemia because malignant cells demand glucose during growth, and patients often lose their appetite [4]. For the effective care of older patients, hypoglycemia should be detected quickly and treated using oral or intravenous glucose.

Hypoglycemia may also be caused by chronic inflammation due to infections in older patients. Chronic infections or transient bacteremia can cause persistent inflammation in the body [5]. Occult bacteremia can also cause hypoglycemia during the clinical course when older patients may not have glucose storage due to sarcopenia and frailty [6]. However, interventions for chronic medical conditions can alter human homeostasis, stimulate various cells, and cause acute inflammation in the body [7]. Here, we report a case of hypoglycemia caused by persistent bacteremia in the biliary tract. Endoscopic retrograde cholangiopancreatography (ERCP) intervention causes a hyperinflammatory status in patients with hyperserotonemia. This case highlights the importance of chronic infection as an etiology of hypoglycemia and the difficulty in deciding on invasive interventions for chronic conditions in older patients.

## Case presentation

An 85-year-old man visited our hospital with chief complaints of progressive anemia and appetite loss. He had visited his family doctor 1 month prior and reported progressive anemia and increased inflammatory findings. He was then referred to our hospital for a full-body examination, including an examination of his residual stomach after gastric cancer surgery. His medical history included dementia, chronic heart failure, benign prostatic hypertrophy, hypertension, and constipation. His drug history included magnesium oxide, furosemide, and tamsulosin.

His vital signs on admission were as follows: blood pressure (BP), 70/50 mmHg; pulse rate, 71 beats/min; respiratory rate, 16 breaths/min; body temperature, 37.7°C; and SpO_2_, 96% (room air). Physical examination revealed pallor of the palpebral conjunctiva. No other specific physical findings were observed on the neck, chest, abdomen, or extremities. Initial laboratory data showed macrocytic anemia and elevated inflammatory markers with high C-reactive protein levels (Table 1).

**Table 1:** Initial laboratory data of the patient PT: prothrombin time; INR: international normalized ratio; APTT: activated partial thromboplastin time; eGFR: estimated glomerular filtration rate; CK: creatine kinase; CRP: C-reactive protein; TSH: thyroid-stimulating hormone; Ig: immunoglobulin; HCV: hepatitis C virus; SARS-CoV-2: severe acute respiratory syndrome coronavirus 2; HIV: human immunodeficiency virus; HBs: hepatitis B surface antigen; HBc: hepatitis B core antigen; C3: complement component 3; C4: complement component 4; KL-6: Krebs von den Lungen-6; MPO-ANCA: myeloperoxidase-antineutrophil cytoplasmic antibodies; anti-SSA/Ro autoantibodies: anti-Sjogren’s syndrome type A autoantibodies; anti-SSB/La autoantibodies: anti-Sjogren syndrome antigen type B autoantibodies; CCP antibodies: cyclic citrullinated peptide antibodies

Marker	Level	Reference
White blood cells	2.70	3.5–9.1 × 10^3^/μL
Neutrophils	63.0	44.0–72.0%
Lymphocytes	23.9	18.0–59.0%
Monocytes	4.7	0.0–12.0%
Eosinophils	5.9	0.0–10.0%
Basophils	02.5	0.0–3.0%
Red blood cells	2.60	3.76–5.50 × 10^6^/μL
Hemoglobin	9.3	11.3–15.2 g/dL
Hematocrit	27.9	33.4–44.9%
Mean corpuscular volume	107.5	79.0–100.0 fL
Platelets	19.3	13.0–36.9 × 10^4^/μL
Total protein	6.5	6.5–8.3 g/dL
Albumin	3.1	3.8–5.3 g/dL
Total bilirubin	0.3	0.2–1.2 mg/dL
Aspartate aminotransferase	26	8–38 IU/L
Alanine aminotransferase	33	4–43 IU/L
Alkaline phosphatase	138	106–322 U/L
γ-Glutamyl transpeptidase	37	<48 IU/L
Lactate dehydrogenase	197	121–245 U/L
Blood urea nitrogen	40.6	8–20 mg/dL
Creatinine	1.82	0.40–1.10 mg/dL
eGFR	28.1	>60.0 mL/min/L
Serum Na	144	135–150 mEq/L
Serum K	4.9	3.5–5.3 mEq/L
Serum Cl	109	98–110 mEq/L
Ferritin	499.1	14.4–303.7 ng/mL
CK	68	56–244 U/L
CRP	0.39	<0.30 mg/dL
TSH	2.45	0.35–4.94 μIU/mL
Free T4	0.9	0.70–1.48 ng/dL
Vitamin B12	511	187–883 pg/mL
IgG	1522	870–1700 mg/dL
Urine test		
Leukocyte	Negative	
Nitrite	Negative	
Protein	Negative	
Glucose	Negative	
Urobilinogen	Normal	
Bilirubin	Negative	
Ketone	Negative	
Blood	Negative	
pH	7.5	
Specific gravity	1.011	

**Figure 1:**
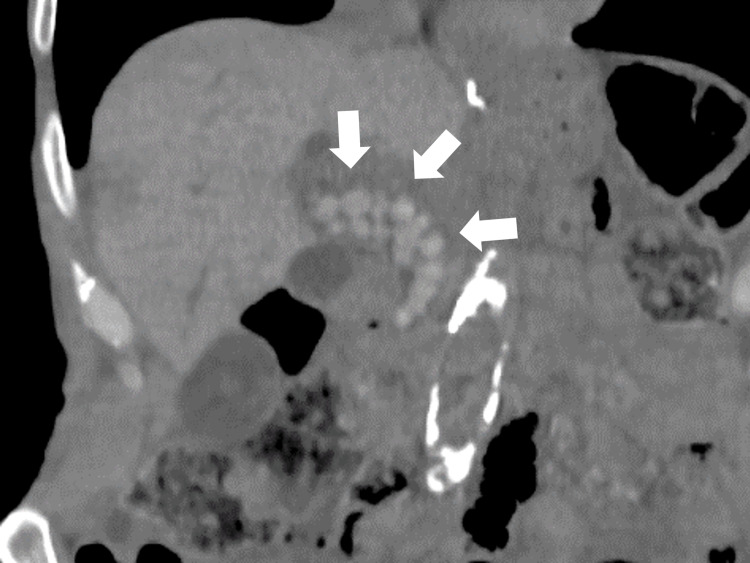
Abdominal computed tomography showing multiple common bile duct stones (white arrows)

On the first day of hospitalization, upper gastrointestinal endoscopy revealed reflux esophagitis. In addition, computed tomography revealed no obvious malignant or inflammatory findings; however, multiple common bile duct stones were found (Figure 1).

Although the patient was administered lansoprazole for reflux esophagitis, anorexia persisted.

On the eighth day of hospitalization, delirium appeared, and laboratory tests revealed hypoglycemia (16 mg/dL), which was treated with an intravenous infusion of 50% glucose. As vital signs were normal without fever, we performed a blood culture test to investigate the possibility of occult bacteremia. On the 10th day of hospitalization, an adrenocorticotropic hormone (ACTH) loading test was performed to investigate adrenal insufficiency. Cortisol was 8.3 μg/dL before ACTH infusion, 12.5 μg/dL 30 min after ACTH infusion, and 12.8 μg/dL 60 min after ACTH infusion, and ACTH was 24 μg/dL. Brain magnetic resonance imaging revealed no mass lesions or inflammatory changes. On the 11th day, the blood culture test revealed the presence of *Klebsiella pneumoniae*. The patient was diagnosed with secondary adrenal insufficiency due to occult bacteremia. He was started on hydrocortisone and intravenous ceftriaxone (2 g/day), and his appetite and state of consciousness improved.

We suspected occult bacteremia caused by multiple chronic common bile duct stones. Based on a discussion with the patient’s family and a gastroenterologist, ERCP was performed on the 29th day of hospitalization to remove the common bile duct stones. On the 32nd day of hospitalization, hepatobiliary enzymes were elevated without additional vital sign changes (aspartate aminotransferase (AST), 245; alanine aminotransferase (ALT), 345). On the 34th day of hospitalization, further elevated hepatobiliary enzymes (AST, 1294; ALT, 634), increased ferritin levels (5090.8 ng/mL), no spherocytosis, and thrombocytopenia (2.2×104/μL) were reported in the patient. The patient was also unconscious with a mild fever during this time. We discussed the need for further investigations with the family, such as bone marrow examination, and they denied the procedure. In addition, the patient could not move to the prone position because of back bending due to osteoporosis and vertebral deformities. Based on fever, hyperferritinemia, high AST, and low platelets, we suspected macrophage activation syndrome (MAS), and methylprednisolone (1000 mg) was started. However, his BP decreased, and the family hoped for palliative care. The patient died on the 39th day of hospitalization.

## Discussion

In this case, multiple chronic common bile duct stones were present, and bacteremia might have been caused by retrograde bacterial infection. The hypoglycemic attack during the treatment course might have been due to secondary adrenal insufficiency caused by bacteremia. Serum ferritin, lactate dehydrogenase, AST, and ALT levels increased after ERCP was performed to remove the common bile duct stones, suggesting MAS.

Hypoglycemia, in this case, might have been caused by critical illness-related corticosteroid insufficiency (CIRCI) from occult bacteremia [8]. CIRCI occurs in patients with severe sepsis [9]. Serum cortisol levels increase after severe sepsis, trauma, or surgery. This hypersecretion triggers cortisol depletion and receptor downregulation, suppressing ACTH and cortisol secretion [9]. This suppression causes decreased cortisol activity during hypoglycemia [8,9]. In this case, an ACTH load test was performed, and post-load cortisol levels did not exceed 18 μg/dL, even in occult bacteremia. In addition, laboratory data showed an extreme drop in serum corticosteroid levels to 12.8 μg/dL after the loading test. Therefore, the condition was diagnosed as secondary adrenal insufficiency. Older patients are more vulnerable to critical conditions than younger patients [9]. Suppression of the adrenocortical axis can be impinged by inflammatory conditions, such as occult bacteremia [9]. In such cases, appetite reduction and hypoglycemia should be investigated using blood culture tests to identify bacteremia.

Interventions for chronic clinical conditions require various considerations, including complications from interventions and subsequent pathophysiology. This patient had multiple common bile duct stones, which might have been the cause of occult bacteremia. On the 29th day of hospitalization, we performed ERCP to remove all common bile duct stones as an intervention for occult bacteremia. However, after the intervention, there was a drastic increase in AST, ALT, and ferritin levels and a decrease in platelets, diagnosed as MAS [10]. MAS is caused by various inflammatory diseases that stimulate macrophages [10]. The pathophysiology of MAS, in this case, might be the activation of Kupffer cells in the liver [11,12]. This patient had multiple chronic common bile duct stones, and the bile duct pressure might have been high. The removal of common bile duct stones by ERCP might have abruptly lowered the intra-bile duct pressure, which can stimulate Kupffer cells in the liver sinusoids [13], causing MAS.

The diagnosis and treatment of MAS are challenging, and the critical point is the suppression of hyperactivated macrophage functions in the blood and bone marrow. Diagnosis can be difficult in critically ill older patients due to the difficulty of performing bone marrow examinations [10,11]. Positioning older patients for bone marrow tests can be challenging due to their frailty. In addition, ageism may impinge on performing bone marrow tests by the refusal of the patients’ families to give consent for the test [14,15]. Steroid pulse therapy can be a treatment option for MAS; however, its effectiveness may be limited in older patients [10]. The fundamental treatment for MAS is the control of MAS-causing acute diseases. However, the etiology may not be controlled, and treatment can be difficult. In our case, the patient could not move to the prone position because of back bending caused by osteoporosis and vertebral deformities. Although the patient was treated with a methylprednisolone pulse, the treatment was ineffective in suppressing inflammation.

## Conclusions

This case shows a drastic clinical course of possible MAS triggered by ERCP in a patient with multiple chronic common bile duct stones. Interventions for chronic conditions may impair homeostasis in human physiology and stimulate macrophages, causing MAS. Interventions should be discussed among frail older patients with chronic conditions, including triggering other acute conditions, such as MAS.
